# The role of mycotoxins in lipid metabolism and homeostasis: a systematic review

**DOI:** 10.3389/ftox.2026.1870680

**Published:** 2026-07-07

**Authors:** Bernadett Csókay, Eszter Ruff, Zsuzsanna Szőke, Levente Sára, Balázs Komoróczy, Apolka Szentirmay, Márkó Unicsovics, Katalin Sára-Popovics, Dóra Holéci, Balázs Hamar

**Affiliations:** 1 Department of Obstetrics and Gynecology, Semmelweis University, Budapest, Hungary; 2 Department of Animal Biotechnology, Institute of Genetics and Biotechnology, Hungarian University of Agriculture and Life Sciences, Gödöllő, Hungary; 3 Juretmed Healthcare Service and R&D Consultant Ltd, Hyderabad, India

**Keywords:** adipose tissue, aflatoxins, bioaccumulaion, deoxynivalenol (DON), lipid metabilism, PPAR signaling, zearalenone (ZEN)

## Abstract

**Background:**

Mycotoxins are widespread food contaminants that pose a significant global health concern. While their hepatotoxic, nephrotoxic, and carcinogenic effects are well established and characterized, their effects on adipose tissue biology and systemic lipid regulation remain incompletely understood. Given adipose tissue’s central role in lipid homeostasis, endocrine regulation, and as a storage reservoir for adipophilic compounds, understanding these effects is critical.

**Methods:**

This systematic review was conducted in accordance with the PRISMA 2020 guidelines, and prospectively registered to PROSPERO (CRD42022287044). A comprehensive literature search was conducted in CENTRAL, Embase, and MEDLINE (PubMed) through 16 January 2026. Experimental *in vivo* and *in vitro* studies examining the effects of mycotoxins on adipose tissue, lipid metabolism, or mycotoxin distribution in fat tissue were included. Risk of bias in animal studies was assessed using the SYRCLE Risk of Bias Tool. A descriptive qualitative synthesis was performed.

**Results:**

Twenty-one experimental studies met the inclusion criteria. Across animal models, mycotoxin exposure consistently disrupted lipid homeostasis, leading to elevated serum triglyceride, cholesterol, LDL, and VLDL levels, alongside reduced HDL concentrations. These effects were mediated through toxin-specific mechanisms, including inhibition of *ppar*α signaling by aflatoxins and ochratoxin, suppression of *pparγ* by deoxynivalenol, and activation of *pparγ* by zearalenone. Mycotoxins also impaired adipokine secretion, with reduced leptin and adiponectin levels, and were associated with insulin resistance and altered glucose metabolism. Importantly, although mycotoxins are primarily metabolized in the liver, multiple studies demonstrated substantial accumulation and prolonged retention in adipose tissue, suggesting a secondary toxicokinetic reservoir.

**Conclusion:**

Mycotoxins exert significant, toxin-specific effects on lipid metabolism and adipose tissue function and may contribute to cardiometabolic dysfunction. Adipose tissue appears to play a key role in mycotoxin bioaccumulation, with potential implications for chronic exposure and delayed systemic toxicity.

## Highlights

- Mycotoxins disrupt lipid metabolism via toxin-specific molecular pathways that involve *ppar*α and *ppar*γ signaling.
- Adipose tissue functions as an active endocrine and toxicokinetic compartment in mycotoxin exposure.
- Several mycotoxins accumulate in fat tissue, potentially serving as a long-term reservoir during chronic or high-level exposure.

## Introduction

Mycotoxins are toxic secondary metabolites produced by filamentous fungi that commonly contaminate staple foods such as cereals, maize, nuts, and animal feed. Consequently, both humans and animals are frequently exposed to these compounds, often chronically and at low doses (Alshannaq and Yu, 2017). Exposure is particularly pronounced in regions with inadequate food storage and weak regulatory control (Falade et al., 2022). Among the most relevant mycotoxins are deoxynivalenol (DON), zearalenone (ZEN), aflatoxins, ochratoxin A (OTA), fumonisins, T-2 toxin, patulin, citrulin enniatins, and beauvericin, all of which exert diverse toxic effects depending on dose, exposure duration, and metabolic context (Alshannaq and Yu, 2017).

The adverse health effects of mycotoxins have been extensively studied with regard to hepatotoxicity, nephrotoxicity, immunotoxicity, reproductive toxicity, and carcinogenesis (Wild and Gong, 2010). In contrast, their effects on adipose tissue and systemic lipid metabolism have received far less attention. This represents an important knowledge gap, as adipose tissue is now recognized as a metabolically active endocrine organ rather than a passive lipid storage depot. It plays a central role in energy homeostasis, lipid trafficking, insulin sensitivity, and inflammatory regulation (Galic et al., 2010). Dysregulation of adipose tissue function is a major contributor to dyslipidemia, insulin resistance, non-alcoholic fatty liver disease (NAFLD), and cardiovascular disease (Hajer et al., 2008).

Accumulating experimental evidence indicates that mycotoxins can disrupt lipid homeostasis through multiple molecular pathway (Han et al., 2025; Jin et al., 2023).

Adipose tissue also exerts important endocrine functions by secreting adipokines, such as leptin and adiponectin, which regulate appetite, insulin sensitivity, glucose metabolism, and systemic inflammation (Tilg and Moschen, 2006). Beyond its role in functional disruption, adipose tissue may also play a significant role in the toxicokinetics of mycotoxins. Due to their lipophilic properties, several mycotoxins have been shown to accumulate in fatty tissue (Zou et al., 2012; Rodríguez-Carrasco et al., 2016).

Despite the growing number of *in vivo* and *in vitro* studies addressing these mechanisms, the available evidence remains fragmented and heterogeneous (Feng et al., 2017; Badr and Naeem, 2019). Studies differ substantially in experimental models, mycotoxin types and doses, exposure durations, and analytical methods (Han et al., 2025; Abd et al., 2018; Salah et al., 2024). Consequently, a comprehensive synthesis of the effects of mycotoxins on lipid metabolism and adipose tissue biology is lacking.

Therefore, the aim of the present systematic review was to synthesize the existing experimental evidence on the effects of mycotoxins on adipose tissue and lipid metabolism, and to evaluate the distribution and accumulation of mycotoxins in fat tissue. By integrating mechanistic, biochemical, and toxicokinetic findings, this review seeks to clarify how mycotoxin exposure may contribute to metabolic dysregulation and increased cardiometabolic risk, particularly under conditions of chronic or high-level exposure.

## Methods

This systematic review was conducted and reported in accordance with the PRISMA 2020 guidelines (see Supplementary) and the Cochrane Handbook for Systematic Reviews of Interventions (Cumpston et al., 2019; Page et al., 2021). The review protocol was prospectively registered in the PROSPERO database (CRD42022287044).

### Eligibility criteria

We included studies investigating **adipose tissue or lipid metabolism** in both *in vivo* and *in vitro* settings. Eligible *in vivo* studies involved humans, mammal models, or avian models. *In vitro* studies using adipocyte cultures or lipid-metabolism-focused experimental systems were also included.

In all eligible studies, the intervention group was exposed to **any type of mycotoxin**, while a comparator group without mycotoxin exposure was required. The primary outcomes were changes in lipid metabolism, and secondary outcomes included the distribution and accumulation of mycotoxins in adipose tissue.

For the primary outcomes related to lipid metabolism, we included studies in which analytical methods were clearly described and, where applicable, the commercial assay kits were specified. Accepted methodologies included polymerase chain reaction, Western blotting, histopathological staining, transcriptomic or genomic sequencing approaches, experimental animal or cell line models, and serum biochemical measurements.

For secondary outcomes assessing mycotoxin distribution in adipose tissue, we included studies that used reliable, validated methods for toxin quantification, such as liquid chromatography coupled with mass spectrometry or fluorescence detection, radiolabeled compounds, and radioactive scanning techniques.

### Information sources

The systematic literature search was conducted on 16 January 2026, across three major electronic databases: the Cochrane Central Register of Controlled Trials (CENTRAL), Embase, and MEDLINE (via PubMed).

### Search strategy

The following key terms were used during the systematic search: *“mycotoxins,” “lipid metabolism,” “fat,”* and *“adipose.”* The complete search strategy is provided in the Supplementary Materials.

### Selection process

Article selection was performed using reference management software (EndNote). Two independent reviewers (BH and BK) conducted duplicate removal, title and abstract screening, and full-text eligibility assessment. Inter-reviewer agreement was evaluated using **Cohen’s kappa coefficient (κ)**. Any disagreements were resolved through consultation with a third reviewer (LS).

### Data collection process

Two independent reviewers (BH and EH) extracted predefined data using a standardized Microsoft Excel spreadsheet (Windows 11 Pro). Extracted study-level data included first author, year of publication, digital object identifier (DOI), study type and design, and country of origin.

For both intervention and control groups, the following variables were collected: mycotoxin type (including ZEN, DON, OTA, aflatoxin, T-2 toxin, citreoviridin, FB1, enniatin, and beauvericin), serum triglycerides, total cholesterol, low-density lipoprotein (LDL), very low-density lipoprotein (VLDL), and high-density lipoprotein (HDL). In addition, data on key regulators of lipid metabolism were extracted, including *pparα* and *c/ebpα*.

### Risk of bias and quality assessment of the included articles

Risk of bias in the included *in vivo* animal studies was assessed using the **SYRCLE Risk of Bias Tool**, which is specifically designed for laboratory animal experiments (Hooijmans et al., 2014). The tool evaluates selection, performance, detection, attrition, reporting, and other sources of bias across ten predefined domains. Each domain was judged to have low, high, or unclear risk of bias. Assessments were performed independently by two reviewers (BH and BK), with disagreements resolved by discussion or consultation with a third reviewer (LS). Risk-of-bias judgments were considered during qualitative synthesis, but were not used as exclusion criteria.

### Synthesis methods

We conducted a descriptive systematic review; no quantitative meta-analysis was performed.

## Results

### Search and selection

**FIGURE 1:**
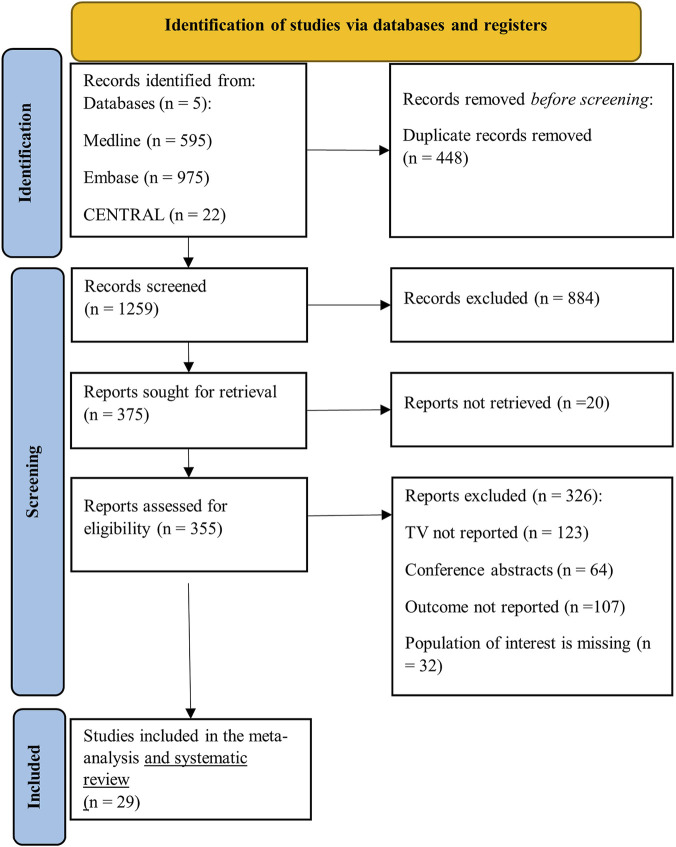
PRISMA 2020 flowchart representing the study selection process.

A total of **6,752 records** were identified through the systematic search. After removal of duplicates, **4237 articles** remained for title and abstract screening. Full-text assessment was conducted for **82 studies**, of which **21 studies** were included in the qualitative synthesis and quality assessment. Cohen’s kappa coefficients were **0.79** for title and abstract screening and **0.84** for full-text selection, indicating substantial agreement between reviewers (Figure 1). Baseline characteristics of the included studies are summarized in Table 1.

**TABLE 1:** Basic characteristics of included studies.

Study	Species	Sex	Mycotoxin	Dose (µg/kg.bw)	Exposure duration	Route	Diet	Outcome
Badr et al., 2019	Rats	Male	AFB1, AFG1	0,85	5 weeks	Oral gavage	Standard	Lipid metabolism
Jin et al., 2023	Mice	Male	DON	100 1,000	4 weeks	Oral gavage	High-fat	Lipid metabolism
Han et al., 2025	Mice	Male	ZEN	50 500	12 weeks	Oral gavage	High-fat	Lipid metabolism
Abdel-Latif et al., 2017	Rats	Male	AFB1	120	8 weeks	Feed-based contamination	Standard	Lipid metabolism
Alm-Eldeen et al., 2017	Rats	Male	AFB1	250	One does	Injection	Standard	Lipid metabolism
Zanganeh et al., 2025	Chicken	Female	AFB1, OTA	2,500 2000	6 weeks	Feed-based contamination	Standard	Lipid metabolism
Salah et al., 2024	Chicken	Female	OTA	1,000	6 weeks	Feed-based contamination	Standard	Lipid metabolism
Zeng et al., 2025	Rat	Male	AFB1, AFB2	20 40	2 weeks	Oral gavage	CDAHFD	Lipid metabolism
An et al., 2024	Duck	Male	T-2	200 400 800	2 weeks	Oral gavage	Standard	Lipid metabolism
Feng et al., 2017	Mice	Male	CIT	300	6 weeks	Intraperitoneally	Standard	Lipid metabolism
Abdel-Wahhab et al., 2018	Rat	Female	DON	5,000	3 weeks	Oral gavage	Standard	Lipid metabolism
El-Nekeety et al., 2017	Mice	Female	FB1 ZEN	50 40	3 weeks	Oral gavage	Standard	Lipid metabolism
Dobrocsyova 2019	Rat adipocyte cells	NA	OTA	500 nmol	2 weeks	NA	NA	Lipid metabolism
Nagl et al., 2021	Pig	Female	ZEN	500 1,500	4 weeks	Feed-based contamination	Standard	Lipid metabolism
Zhao et al., 2020	Mice	Male	DON	300	4 weeks	Gavage	Standard AIN-93M	Lipid metabolism
Rodriguez-Carrasco et al., 2016	Mice	Male	Enniatins, beauvericin	5,000	3–4 days	Intraperitoneally	Standard	Lipid distribution
Castell et al., 2024	Human	Male/female	Enniatins, beauvericin	NA	Autopsy	NA	NA	Lipid distribution
Fodor J et al., 2008	Piglet	NA	FB1 FB2 FB3	45,000 8,600 4,600	8weeks	Feed-based contamination	Standard	Lipid distribution
Kane et el 1986	Rat	Male	OTA	288	2 days	Feed-based contamination	Standard	Lipid distribution
Prelusky et al., 1986	Chicken	Female	DON	2,200	6 days	Feed-based contamination	Standard	Lipid distribution
Shin 2009	Rats	Male	ZEN	1,000 2000 4,000 8,000	1 dose	Intravenous per os	Standard	Lipid distribution

Abbreviations: AFB1, aflatoxin B1; AFB2, aflatoxin B2; AFB3, aflatoxin B3; AFG1, aflatoxin G1; CIT, citrinin; DON, deoxynivalenol; FB1, fumonisin B1; FB2, fumonisin B2; FB3, fumonisin B3; OTA, ochratoxin A; T-2, T-2 toxin; ZEN, zearalenone; AIN-93M, American Institute of Nutrition maintenance diet (standard rodent diet); CDAHFD, choline-deficient L-amino acid-defined high-fat diet; bw, body weight; i. p., intraperitoneal; NA, not available or not reported.

### Mycotoxins and lipid metabolism

#### Effects of mycotoxins on serum lipid and lipoprotein levels

##### Triglyceride levels in rodent models

**FIGURE 2:**
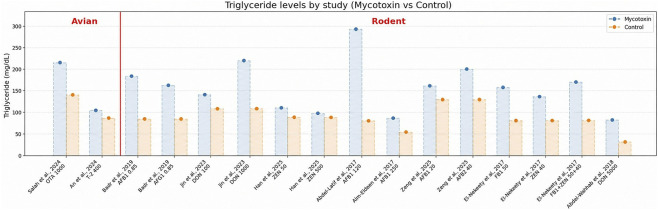
Triglyceride levels by study. This point-based comparative plot shows study-level triglyceride concentrations in mycotoxin-exposed and control groups. Each point represents the mean value reported in the original study. Studies are grouped by species, with avian models on the left and rodent models on the right. The red vertical line separates the two species groups. The x-axis includes author, year, mycotoxin type, and administered dose (µg/bw.kg). Values are expressed in mg/dL. Abbreviations: TG, triglycerides; AFB1, aflatoxin B1; AFG1, aflatoxin G1; DON, deoxynivalenol; ZEN, zearalenone; OTA, ochratoxin A; FB1, fumonisin B1; T-2, T-2 toxin.

Eight studies reported triglyceride levels in rodent models. In all studies, triglyceride levels were reported to be higher in the mycotoxin-exposed groups than in the corresponding control groups (Han et al., 2025; Jin et al., 2023; Badr and Naeem, 2019; Abd et al., 2018; Abdel-Latif et al., 2017; Alm-Eldeen et al., 2017; Zeng et al., 2025; El-Nekeety et al., 2017). The investigated mycotoxins included DON, ZEN, AFB1, AFB2, AFG1, and FB1 (Figure 2). Exposure to FB1 and ZEN demonstrated combined disruptions in triglyceride levels (El-Nekeety et al., 2017).

##### Triglyceride levels in avian models

Two studies evaluated triglyceride levels in avian models exposed to mycotoxins (Salah et al., 2024; An et al., 2024). Both studies reported higher triglyceride levels in the exposed groups compared with controls. The investigated mycotoxins were OTA and T-2 toxin (Figure 2).

##### Cholesterol levels in rodent models

**FIGURE 3:**
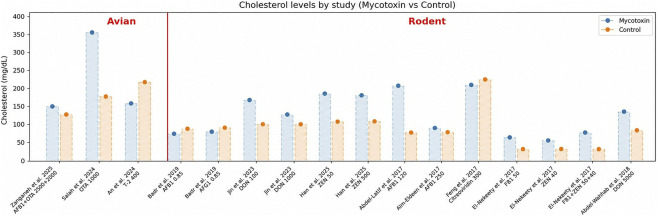
Total cholesterol levels by study. This point-based comparative plot presents study-level total cholesterol concentrations in mycotoxin-exposed and control groups. Each point indicates the mean value reported in the original study. Studies are arranged by species, with avian models displayed on the left and rodent models on the right. The red vertical line marks the separation between species groups. The x-axis shows author, year, mycotoxin type, and administered dose (µg/bw.kg) Values are expressed in mg/dL. Abbreviations: TC, total cholesterol; AFB1, aflatoxin B1; AFG1, aflatoxin G1; DON, deoxynivalenol; ZEN, zearalenone; OTA, ochratoxin A; FB1, fumonisin B1; T-2, T-2 toxin.

Eight studies reported cholesterol levels in rodent models (Han et al., 2025; Jin et al., 2023; Feng et al., 2017; Badr and Naeem, 2019; Abd et al., 2018; Abdel-Latif et al., 2017; Alm-Eldeen et al., 2017; Zeng et al., 2025; El-Nekeety et al., 2017). Five of these studies described higher cholesterol levels in the mycotoxin-exposed groups compared with controls. The investigated mycotoxins included DON, ZEN, AFB1, FB1, AFG1, and citreoviridin. The study investigating citreoviridin reported comparatively moderate alterations in serum lipid profiles (Figure 3).

##### Cholesterol levels in avian models

Three studies investigated cholesterol levels in avian models (Salah et al., 2024; An et al., 2024; Zanganeh et al., 2025). These studies reported higher cholesterol levels following exposure to OTA, T-2 toxin, and AFB1 compared with control groups (Figure 3).

##### LDL levels in rodent models

**FIGURE 4:**
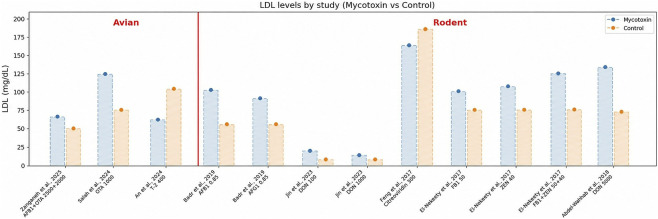
LDL levels by study. This point-based comparative plot shows study-level LDL cholesterol concentrations in mycotoxin-exposed and control groups. Each point represents the mean LDL value reported in the original study. Studies are grouped by species, with avian models on the left and rodent models on the right. The red vertical line indicates the boundary between species groups. The x-axis includes author, year, mycotoxin type, and administered dose (µg/bw.kg). Values are expressed in mg/dL. Abbreviations: LDL, low-density lipoprotein AFB1, aflatoxin B1; AFG1, aflatoxin G1; DON, deoxynivalenol; ZEN, zearalenone; OTA, ochratoxin A; FB1, fumonisin B1; T-2, T-2 toxin.

Five studies reported LDL levels in rodent models exposed to mycotoxins (Jin et al., 2023; Feng et al., 2017; Badr and Naeem, 2019; Abd et al., 2018; El-Nekeety et al., 2017). Four studies reported higher LDL levels in the mycotoxin-exposed groups compared with controls. The investigated mycotoxins included DON, ZEN, AFB1, AFG1, FB1, and citreoviridin. The most moderate changes were observed in the citreoviridin-exposed group (Figure 4).

##### LDL levels in avian models

Three studies reported LDL levels in avian models (Salah et al., 2024; An et al., 2024; Zanganeh et al., 2025). Two studies reported higher LDL levels following exposure to AFB1 and OTA, whereas one study reported lower LDL levels following exposure to T-2 toxin (Figure 4).

##### HDL levels in rodent models

**FIGURE 5:**
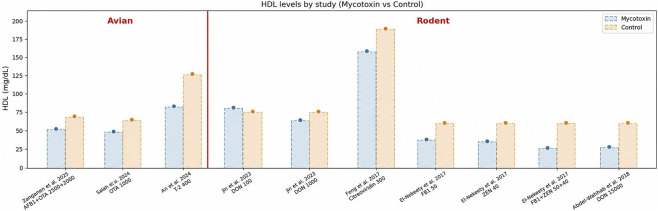
HDL levels by study. This point-based comparative plot presents study-level HDL cholesterol concentrations in mycotoxin-exposed and control groups. Each point indicates the mean HDL value reported in the original study. Studies are grouped by species, with avian models displayed on the left and rodent models on the right. The red vertical line separates the two species groups. The x-axis shows author, year, mycotoxin type, and administered dose (µg/bw.kg). Values are expressed in mg/dL. Abbreviations: HDL, high-density lipoprotein; AFB1, aflatoxin B1; AFG1, aflatoxin G1; DON, deoxynivalenol; ZEN, zearalenone; OTA, ochratoxin A; FB1, fumonisin B1; T-2, T-2 toxin.

Four studies reported HDL levels in rodent models (Jin et al., 2023; Feng et al., 2017; Abd et al., 2018; El-Nekeety et al., 2017). In studies investigating DON, ZEN, citreoviridin, and FB1, HDL levels were generally lower in the mycotoxin-exposed groups than in controls. However, one study using a lower dose of DON did not report a reduction in HDL levels (Figure 5).

##### HDL levels in avian models

Three studies reported HDL levels in avian models (Salah et al., 2024; An et al., 2024; Zanganeh et al., 2025). Exposure to AFB1, OTA, and T-2 toxin was associated with lower serum HDL levels compared with controls (Figure 5).

##### DON and dysregulation of lipid homeostasis in rodent models

Multiple studies demonstrated that mycotoxin-induced dyslipidemia arises through complex, toxin-specific mechanisms. During fasting, circulating triglycerides are transported in very low-density lipoprotein (VLDL) particles synthesized in the liver. However, data showed that hepatic expression of key lipogenic regulators at both the transcriptional and protein levels (*fasn, acc, srebp-1c, pparγ, dgat2*), as well as proteins involved in VLDL assembly (*apob100*, *mtp*), remained unchanged or decreased in DON-exposed mice. These findings indicate that elevated circulating triglyceride levels are not driven by increased hepatic lipid production (Jin et al., 2023). Further studies demonstrated that DON exposure downregulated *ppar*γ expression, leading to reduced lipoprotein lipase activity and impaired free fatty acid uptake in adipose tissue. This ultimately led to triglyceride accumulation in both serum and liver (Jin et al., 2023; An et al., 2024). In addition, DON inhibited adipogenesis by reducing *ppar*γ and *c/ebpα* expression, thereby suppressing genes involved in adipocyte differentiation and lipid storage. Consequently, adipose tissue capacity for fatty acid storage was markedly impaired (Zhao et al., 2020). Moreover, DON increased the expression of lipolytic enzymes, including hormone-sensitive lipase and adipose triglyceride lipase, thereby promoting enhanced lipolysis and further elevating circulating triglyceride levels (Jin et al., 2023).

##### ZEN and dysregulation of lipid homeostasis in rodent models

In contrast, ZEN exposure upregulated *ppar*γ signaling and hepatic lipid synthesis, resulting in increased circulating lipid levels (Han et al., 2025; Li et al., 2014). Lipogenic transcription factors, including *ppar*γ and *srebf*1, were activated, leading to increased expression of lipid synthesis genes, including *fasn* and *hmgcr*. The divergent metabolic effects of DON and ZEN were particularly evident in high-fat diet models: DON-treated mice exhibited weight loss due to impaired triglyceride uptake and storage in adipose tissue, whereas ZEN-treated mice demonstrated increased weight gain driven by enhanced hepatic lipogenesis with relatively preserved adipose tissue function (Han et al., 2025; Jin et al., 2023).

##### Aflatoxin and dysregulation of lipid homeostasis in rodent models

During aflatoxin exposure, hepatic *pparα* signaling was inhibited, resulting in impaired lipoprotein lipase activity and reduced fatty acid uptake. Two studies employing aflatoxin infusion in rats confirmed this mechanism by demonstrating downregulation of *ppar*α and its target genes involved in fatty acid oxidation, reverse cholesterol transport, and lipoprotein remodeling (*cpt1, lcat, scarb1, lipc*) (Zeng et al., 2025; Rotimi et al., 2017). In contrast, genes promoting fatty acid and triglyceride synthesis (*srebp1c, fasn, acc*) were upregulated in the liver (Zeng et al., 2025).

##### OTA, T2, and dysregulation of lipid homeostasis in avian models

Similarly, in poultry models exposed to OTA, elevated serum levels of VLDL, triglycerides, and cholesterol were observed, likely reflecting inhibition of hepatic *pparα* signaling (Salah et al., 2024). T-2 toxin exposure also suppressed the *pparα* pathway, leading to dyslipidemia and reduced weight gain (An et al., 2024).

##### OTA, ZEN on endocrine signaling of adipose tissue

In adipocyte cell culture models, exposure to OTA impaired adipogenesis, as evidenced by reduced Oil Red O staining and decreased adipocyte differentiation. Key adipose-derived endocrine regulators, including leptin and adiponectin, were significantly reduced in OTA-treated cells (Dobrocsyova et al., 2019). Similarly, studies investigating ZEN exposure reported decreased levels of leptin and adiponectin (Nagl et al., 2021). Mycotoxin exposure was also associated with increased production of reactive oxygen species and proinflammatory cytokines, which may further disrupt adipokine signaling and adipose tissue endocrine function (Dobrocsyova et al., 2019; Nagl et al., 2021).

##### DON, ZEN, aflatoxin on glucose metabolism in adipose tissue

Five studies investigated alterations in glucose metabolism associated with mycotoxin exposure (Han et al., 2025; Jin et al., 2023; Abdel-Latif et al., 2017; Dobrocsyova et al., 2019; Nagl et al., 2021). In all studies involving DON, ZEN, or aflatoxin exposure, serum glucose levels were elevated compared with control groups in rodent models (Han et al., 2025; Jin et al., 2023; Abdel-Latif et al., 2017). In one study examining DON exposure, oral glucose tolerance testing revealed insulin resistance and impaired glucose tolerance, particularly in animals receiving a high-fat diet (Jin et al., 2023). In contrast, ZEN exposure did not consistently induce insulin resistance or impaired glucose tolerance (Han et al., 2025). In adipocyte culture studies exposed to OTA, elevated glucose concentrations were accompanied by reduced expression of glucose transporter 4 (GLUT4) and insulin receptor substrate 1 (IRS1), indicating impaired insulin-mediated glucose uptake in adipose cells (Dobrocsyova et al., 2019). Another study with pigs suggested that ZEN-induced alterations in adiponectin signaling may contribute to dysregulation of insulin signaling pathways and glucose metabolism (Nagl et al., 2021).

##### Mycotoxins and tissue distribution in adipose tissue

Six studies investigated the distribution of mycotoxins in adipose tissue (Rodríguez-Carrasco et al., 2016; Shin et al., 2009; Fodor et al., 2008; Kane et al., 1986; Prelusky et al., 1986; Castell et al., 2024). In one human autopsy study, enniatin and beauvericin were detected in multiple tissues, including adipose tissue, liver, brain, heart, and kidney. The highest incidence and concentrations were observed in liver tissue; however, adipose tissue demonstrated the highest co-occurrence rate, with all five tested mycotoxins (four enniatin subtypes and beauvericin) present in **79%** of samples—the highest among all tissues analyzed (Castell et al., 2024).

In a rat study, the distribution of beauvericin and enniatin revealed that both mycotoxins accumulated predominantly in the liver, with adipose tissue representing the second-highest concentration site. Beauvericin exhibited greater stability and higher adipose tissue concentrations than enniatin, which underwent phase I metabolism in the liver and colon. The slower metabolism of beauvericin may account for its increased toxic potential (Rodríguez-Carrasco et al., 2016).

Another rat study examining ZEN infusion demonstrated high concentrations of ZEN in adipose tissue, as well as in the liver, kidney, and small intestine—organs involved in toxin metabolism and excretion (Shin et al., 2009). In piglets exposed to FB1, moderate concentrations were detected in adipose tissue, while the highest levels were observed in the liver and kidney (Fodor et al., 2008).

Notably, one study employing radiolabeled OTA in rats demonstrated progressive accumulation in adipose tissue over time. OTA concentrations increased at 5, 24, and 48 h post-exposure, with adipose tissue exhibiting the highest levels at 48 h. In contrast, concentrations in other organs declined over time, indicating active excretion, whereas adipose tissue showed sustained accumulation consistent with bioaccumulation (Kane et al., 1986). Similarly, a study using radiolabeled DON in avian models demonstrated accumulation in adipose tissue up to 96 h following exposure (Prelusky et al., 1986).

##### Risk of bias assessment and quality of evidence

Overall, most included animal studies demonstrated low risk of bias across most SYRCLE domains, indicating generally good methodological quality. Domains related to outcome assessment and completeness of outcome data were consistently judged to be at low risk of bias. Some domains were rated as **unclear risk**, mainly due to insufficient reporting of randomization or blinding rather than evident methodological shortcomings. Overall, the quality of the included studies was considered acceptable, and the evidence was deemed suitable for qualitative synthesis (see Supplementary).

## Discussion

We investigated the effects of mycotoxins on adipose tissue and lipid metabolism. In rodent and avian models, mycotoxin exposure was consistently associated with elevated serum lipid and lipoprotein levels. These effects appear to be mediated primarily by negative regulation of ***pparα*** in rodents and avians. In rodent models, ***pparγ*** was negatively regulated by DON, whereas zearalenone (ZEN) was found to increase *pparγ* activity. In addition, mycotoxins reduced circulating leptin and adiponectin levels and were associated with impaired glucose tolerance and insulin resistance. We further examined the distribution of mycotoxins in adipose tissue. Although mycotoxins are primarily metabolized in the liver, our findings indicate that they reach high concentrations in adipose tissue, where they appear to accumulate for longer periods than in other organs. This prolonged retention suggests that adipose tissue may serve as a secondary reservoir for mycotoxins following systemic exposure.

Available preclinical evidence suggests that certain mycotoxins may affect lipid metabolism through partly overlapping but toxin-specific mechanisms. DON has been particularly well studied in the context of lipid homeostasis and energy metabolism. DON exposure has been shown to induce anorexia, weight loss, and a reduction in adipose tissue mass in mice (Amuzie et al., 2011). One study demonstrated that DON-induced anorexia is accompanied by activation of the central nervous system and increased catecholamine release, leading to enhanced lipolysis and elevated circulating free fatty acid levels (Barbouche et al., 2020). This, in turn, promotes hepatic lipid accumulation and contributes to the development of steatohepatitis or non-alcoholic fatty liver disease (NAFLD), as both lipogenesis and lipolysis are dysregulated in the liver (Jin et al., 2023; Barbouche et al., 2020).

These experimental findings indicate that mycotoxin exposure can alter lipid-related pathways and circulating lipoprotein profiles in preclinical models. Such changes are biologically relevant because dyslipidemia is a recognized cardiovascular risk factor; however, the present evidence does not establish that mycotoxin exposure causes cardiovascular disease or atherosclerosis in humans (Garg et al., 2015). In many studies, mycotoxin exposure was associated with decreased high-density lipoprotein (HDL) levels. Given HDL’s role in reverse cholesterol transport, reduced HDL concentrations are indicative of increased cardiovascular risk (Ouimet et al., 2019). Moreover, low-density lipoprotein (LDL) levels were elevated in many studies, a change strongly associated with endothelial plaque formation and an elevated risk of cardiovascular and neurological diseases (Ference et al., 2017). Experimental studies suggest that selected mycotoxins can induce oxidative stress, lipid peroxidation, and inflammatory responses, which are mechanistically related to vascular injury. Nevertheless, direct evidence that these mechanisms translate into clinically relevant atherosclerosis in humans remains insufficient (Malvandi et al., 2022).

Mycotoxins have also been associated with hepatocellular injury in preclinical models. Several studies have demonstrated that mycotoxin-induced liver injury is mediated through ferroptosis, driven by increased reactive oxygen species production, depletion of antioxidant peroxidase systems, and uncontrolled necrotic cell death (Abdel-Wahhab et al., 2015). In addition, AFB1 has been shown to induce necroptosis, a regulated but non-apoptotic form of cell death mediated by receptor-interacting protein (RIP) kinases rather than caspases (Chen et al., 2019). These pathways are mechanistically relevant to hepatic inflammation and metabolic liver injury; however, the current evidence does not establish that mycotoxin exposure accelerates NAFLD or NASH progression in humans (Zeng et al., 2025). Rather, the available data suggest that mycotoxins may aggravate hepatic cellular stress and lipid-handling abnormalities under experimental conditions, particularly in models involving high-dose exposure or metabolic stress (Jin et al., 2023; Dopavogui et al., 2023).

A limitation of our study is that several included studies used high-dose or acute exposure models. Therefore, it remains difficult to distinguish direct mycotoxin-mediated dysregulation of lipid metabolism from secondary metabolic alterations caused by hepatic injury and subsequent necrosis. Consequently, the observed changes in serum lipid levels may reflect the combined effects of both mechanisms.

Notably, in most experimental studies, administered mycotoxin doses were approximately 100–1000 times higher than typical human exposure levels. Consequently, the direct translational relevance of these findings to everyday human exposure is limited, as the high doses used in many animal models are unlikely to reflect real-life exposure scenarios, even in the context of severe contamination events or mycotoxicosis outbreaks. Although exceptionally high mycotoxin levels may occur in specific regions or outbreak settings, these concentrations generally remain substantially lower than those administered in experimental animal studies. In addition, food-borne mycotoxin levels are routinely monitored and regulated by food safety authorities in many countries, further reducing the likelihood of exposure to such extreme doses under normal regulatory conditions (Falade et al., 2022; Zhou et al., 2023). Thus, the high doses used in several experimental studies are unlikely to represent typical human exposure under regulated conditions. However, exposure may still be underestimated in settings with limited food-safety control, poor storage conditions, multiple mycotoxin co-exposure, or masked mycotoxins (Freire and Sant’Ana, 2018). Under such extreme exposure conditions, experimental studies have shown that supplementation with antioxidant substrates such as chlorophyllin, coumarin, silymarin, and inulin can partially reverse mycotoxin-induced hepatotoxicity and lipid metabolic disturbances in rodents (Abd et al., 2018; Abdel-Latif et al., 2017). In regions affected by chronic mycotoxin contamination, the safe and effective use of antioxidant interventions may therefore warrant further consideration. Moreover, biological adsorbents and nutritional extracts, such as yeast cell wall extract, have recently been shown to mitigate the combined toxic effects of aflatoxin B1 and deoxynivalenol on hepatic health, intestinal integrity, oxidative stress, and gut microbiota composition, suggesting their potential value as practical feed-based interventions in mycotoxin-contaminated settings (Yu et al., 2025).

Mycotoxins exhibit diverse toxicokinetic profiles, strongly influenced by their chemical structure, lipophilicity, and metabolic stability. Following oral exposure, most mycotoxins are rapidly absorbed in the gastrointestinal tract and distributed primarily to metabolically active organs, particularly the liver and kidney, where biotransformation and excretion occur (Kőszegi and Poór, 2016). Hepatic phase I and II metabolism plays a central role in detoxification; however, incomplete metabolism or the formation of bioactive metabolites may prolong systemic exposure (Ropejko and Twarużek, 2021). While elimination via bile or urine is efficient for several mycotoxins, increasing evidence indicates that certain compounds are redistributed into peripheral tissues, including adipose tissue, where clearance is slower (Shin et al., 2009; Kane et al., 1986).

The present findings suggest that adipose tissue represents a relevant secondary compartment in mycotoxin toxicokinetics. Lipophilic mycotoxins such as enniatins, beauvericin, ZEN, and OTA tend to accumulate in fat tissue, in some cases exceeding concentrations observed in other organs over time. Radiolabeled studies provide particularly strong evidence for true bioaccumulation, demonstrating declining toxin levels in classical metabolizing organs alongside progressive accumulation in adipose tissue. This kinetic pattern supports the concept that adipose tissue acts as a long-term reservoir, with the potential to delay toxin release during periods of increased lipolysis, such as fasting, weight loss, pregnancy, or metabolic stress. Adipose tissue should therefore be considered a dynamic toxicokinetic compartment rather than a passive storage depot. Initial sequestration of relatively lipophilic mycotoxins in adipose tissue may theoretically reduce the circulating free toxin fraction and thereby provide a short-term buffering effect against acute systemic toxicity. However, this storage may also prolong internal exposure. During physiological or pathological states associated with increased lipolysis, such as fasting, severe negative energy balance, lactation, rapid weight loss, or metabolic stress, adipose-stored lipophilic compounds may theoretically be remobilized into the circulation and contribute to delayed or secondary systemic exposure. Nevertheless, direct evidence for clinically relevant remobilization remains limited and is likely to be highly toxin-specific, depending on lipophilicity, protein binding, metabolism, and tissue distribution. Future toxicokinetic studies should therefore evaluate mycotoxin behavior under dynamic metabolic conditions rather than only under steady-state feeding conditions (La Merrill et al., 2013; Jansen et al., 2017).

Due to global warming and global grain trade, mycotoxins have also appeared (e.g., aflatoxins in the Carpathian basin) that were not previously typical of the given region (Casu et al., 2024). Furthermore, obesity, typical of Western societies, increases the incidence of certain diseases, including diabetes, insulin resistance, infertility, and endometrial cancer (Szőke et al., 2025; Szentirmay et al., 2024; Unicsovics et al., 2024). In addition to direct effects, the prolonged mycotoxin storage capacity of adipose tissue may also contribute to these processes.

## Conclusion

This review shows that mycotoxins disrupt lipid metabolism and adipose tissue function through toxin-specific metabolic pathways and lipophilic mycotoxins accumulate in fat tissue as a secondary toxicokinetic compartment. Adipose tissue may therefore act as a long-term reservoir, potentially contributing to metabolic dysregulation during chronic low-level or high-level mycotoxin exposure.

## Glossary

ACC: acetyl-CoA carboxylase
AFB1: aflatoxin B1
AFB2: aflatoxin B2
AFB3: aflatoxin B3
AFG1: aflatoxin G1
AIN-93M: American Institute of Nutrition maintenance diet (standard rodent diet)
APOB100: apolipoprotein B100
bw: body weight
C/EBPα: CCAAT/enhancer-binding protein alpha
CDAHFD: choline-deficient L-amino acid-defined high-fat diet
CENTRAL: Cochrane Central Register of Controlled Trials
CIT: citrinin
CREdiT: Contributor Roles Taxonomy
DGAT2: diacylglycerol O-acyltransferase 2
DON: deoxynivalenol
FB1: fumonisin B1
FB2: fumonisin B2
FB3: fumonisin B3
FASN: fatty acid synthase
HDL: high-density lipoprotein
HMGCR: 3-hydroxy-3-methylglutaryl-CoA reductase
i.p.: intraperitoneal
IRS1: insulin receptor substrate 1
κ: Cohen’s kappa coefficient
LDL: low-density lipoprotein
LCAT: lecithin–cholesterol acyltransferase
LIPC: hepatic lipase
MEDLINE: Medical Literature Analysis and Retrieval System Online
MTP: microsomal triglyceride transfer protein
NA: not available/not reported
NAFLD: non-alcoholic fatty liver disease
NASH: non-alcoholic steatohepatitis
OTA: ochratoxin A
PPARα: peroxisome proliferator-activated receptor alpha
PPARγ: peroxisome proliferator-activated receptor gamma
PRISMA: Preferred Reporting Items for Systematic Reviews and Meta-Analyses
RIP: receptor-interacting protein (kinases)
ROS: reactive oxygen species
SCARB1: scavenger receptor class B type 1
SREBF1: sterol regulatory element-binding transcription factor 1
SREBP-1c: sterol regulatory element-binding protein 1c
SYRCLE: Systematic Review Centre for Laboratory animal Experimentation
T-2: T-2 toxin
VLDL: very low-density lipoprotein
ZEN: zearalenone
